# Basedow-Graves’ disease in a pediatric patient with Sticlker syndrome, a new endocrine finding to improve personalized treatment

**DOI:** 10.1186/s13052-020-00945-x

**Published:** 2020-12-01

**Authors:** Roberta Onesimo, Cristina De Rose, Clelia Cipolla, Silvia Della Casa, Chiara Leoni, Annabella Salerni, Daniela Ricci, Giuseppe Zampino

**Affiliations:** 1 Center for Rare Diseases and Birth Defects, Department of Woman and Child Health and Public Health, Fondazione Policlinico Universitario A. Gemelli, IRCCS, Rome, Italy; 2 Division of Pediatric, Department of Woman and Child Health and Public Health, Fondazione Policlinico Universitario A. Gemelli, IRCCS, Rome, Italy; 3 Division of Endocrinology and Metabolic Diseases, Catholic University of the Sacred Heart, Rome, Italy; 4 Catholic University of the Sacred Heart, Rome, Italy; 5 Division of Ophthalmology, Fondazione Policlinicico Universitario A. Gemelli, IRCSS, Roma, Italy; 6 Pediatric Neurology Unit, Catholic University of Sacred Heart, Fondazione Policlinico Agostino Gemelli IRCCS, Rome, Italy; 7 National Centre of Services and Research for the Prevention of Blindness and Rehabilitation of Low Vision Patients - IAPB Italia Onlus, Rome, Italy

**Keywords:** Stickler syndrome, Basedow-Graves’s disease, Pediatric disability, Autoimmune disease, Nutritional assessment

## Abstract

**Background:**

Stickler syndrome is a connective tissue disorder with predominantly autosomal dominant inheritance, with ocular, auditory and joint involvement.

Thyroid dysfunction was not described as part of alterations in Stickler syndrome and in particular, the association between Stickler’s syndrome and Graves’ disease has never been previously reported in literature.

Moreover, the presence of Graves’ disease is uncommon in the pediatric age (especially in children younger than 6 years old).

**Case presentation:**

We report the case of a 5-years old child affected by Stickler syndrome who received the diagnosis of Graves’s disease, in absence of suggestive symptoms, during health supervision.

**Conclusions:**

This is the first evidence of thyroid dysfunction and autoimmune pattern for Sticker syndrome.

Further clinical reports are expected before suggesting the implementation of new clinical skills for Stickler syndrome, but this paper may contribute to improve personalized management of this rare disorder.

## Introduction

Stickler syndrome (SS) is a rare genetically heterogeneous disorder of the connective tissue, caused by abnormal synthesis of type II, XI, or IX collagen [1]. We distinguish different syndrome subtypes: type I, the most common, due to mutations in the COL2A1 gene; type II, due to mutation of the COL11A1 gene; type III caused by mutations in the COL11A2 gene. These three subtypes exhibit an autosomal dominant mode [1]. Autosomal recessive inheritance are associated with mutations in the COL9A1 gene and LOXL3 gene. Penetrance is complete, but phenotypic variability occurring both within and among families.

The full list of genes responsible for SS and associated phenotypes include: COL2A1, COL11A1, COL11A2, COL9A1, COL9A2, COL9A3, LOXL3 and PLOD3.

This variability makes the diagnostic approach difficult and for this reason the incidence of the syndrome can be significantly underestimated, approximately one in 7500–9000 neonates [2].

The typical clinical manifestations of SS are: I) ocular involvement with findings of congenital or early onset cataract, congenital abnormalities of the vitreous humour, retinal detachment and myopia greater than 3 diopters; II) sensorineural or conductive hearing loss; III) midfacial underdevelopment, and cleft palate (as part of the Pierre Robin sequence); IV) joint involvement with mild spondyloepiphyseal dysplasia, joint hypermobility and early onset arthritis [2].

Patients with the syndrome - especially COL11A2 gene mutation - may exhibit short stature.

Association between Stickler’s syndrome and Basedow-Graves’ disease or other thyroid dysfunction has never been reported in literature.

Basedow-Graves’s disease, hypothyroidism and autoimmune thyroiditis with early onset were already described in other genetic syndromes such as Turner syndrome (TS) [3] and Down syndrome (DS) [4, 5].

Basedow-Graves’ disease is uncommon in the pediatric age (especially in children younger than 6 years old) and associated with developing complications due to a possible late diagnosis [6].

The initial clinical presentation is often severe, especially if the child is young and/or pre-pubescent.

We report the case of a 5-years old child affected by type I Stickler syndrome, in which the diagnosis of Graves’s disease was performed in preclinical state during health supervision and general nutritional assessment [7].

## Case presentation

This is a girl affected by Stckler’s syndrome, with clinical and molecular confirmation (mutation c872G > T, p.Gly291Val of COL2A1 gene) in follow up at the Rare Diseases Unit of Fondazione Policlinico Universitario A. Gemelli - IRCCS. The syndrome has maternal segregation.

At 5 years her weight, height, and head circumference were at 10th, 5th percentile and 50th percentile, respectively. Neurological and behavioral assessment did not reveal abnormalities. Eye examination revealed moderate myopia (8/10 diopters). Her hearing responses were intact. The tympanogram showed a slight depression and mild asymmetry of the cocleo-stapedial reflexes. She presented a mild generalized joint hypermobility.

Her vital signs were normal without signs of seizures, tremor, or psychomotor abnormalities. No hepatomegaly, splenomegaly were found.

For short stature, bone age was detected and resulted equivalent to her chronologic age. Laboratory studies revealed a normal levels of hemoglobin concentration, leukocyte and platelet count, aspartate aminotransferase and alanine aminotransferase levels.

Her thyroid-stimulating hormone (TSH) level was < 0.01 microIU/mL (normal range, 0.5–4.8 μIU/mL), free thyroxine (free T4) was 30.3 pg/ml (vn 8.5–16. 5). Thyroid ultrasound showed for a picture of thyroid glandular hyperfunctions (increase of glandular size, inhomogeneous structure and increase of blood flow).

The patient was referred to the Endocrine Pediatric Unit of our Polyclinic for the appropriate appraisals and treatments. The tests confirmed the diagnosis of Basedow-Graves’ disease [TSH < 0.01 microUI / ml; triiodothyronine (T3) 19.9 pg/mL; FT4 42.1 pg/mL; Anti-thyroperoxidase antibodies: > 1300 IU / mL (vn < 60); Anti-receptor antibodies of TSH (TRAb): 13.97 IU/L (vn < 1.75)].

Screening for other autoimmune diseases was performed (total IgA and antitransglutaminase antibodies to exclude celiac disease; GAD autoantibodies (GADA) to exclude type I diabetes mellitus) and it was negative.

As indicated by the paediatric endocrinology consultant, she was treated with Methimazole (MMI) with an initial dose of a half a 5 mg tablet three times a day (0.45 mg/kg/day). Other drugs, such as β-blockers, were not added in absence of cardiological signs or symptoms, according to the literature [8].

After 5 days of therapy with good tolerance, the child was discharged and a short-term endocrinological follow-up was scheduled. Dose scaling is currently on going in reason of the progressive normalization of thyroid function.

## Discussion and conclusions

Graves’s disease (GD) is the commonest cause of hyperthyroidism, 84% of paediatrics cases. It is a rare disease, especially in childhood, accounting for 1 to 5% of all patients with GD. It may occur at any age during childhood, but its frequency increases with age, peaking during adolescence. Its incidence is estimated around 0.1 cases per 100.000 individuals in prepubertal children and 3 cases per 100.000 during adolescence [4].

GD is a form of autoimmune hyperthyroidism where the thyroid is stimulated to produce excess hormones by TSH receptor antibodies (TRAb) [9]. Other more specific signs are thyromegaly, and exophthalmos, that is detectable in only 20% of cases, whilst it is generally considered as a typical clinical sign of GD in adulthood [4, 9]. Ocular signs are generally less severe in children than in adults and can be attributed to the inflammation and muscle swelling rather than to infiltrative disease of the orbital structures [4, 10]. As expected, these signs (lid retraction and eye proptosis) may spontaneously improve in the majority of children after euthyroidism restoration [4, 10]. In addition, pretibial myxedema is more infrequent in children than in adults [4, 10]. Typical of pediatric GD is the acceleration of linear growth and bone maturation associated with prolonged hyperthyroidism [4, 11]. These clinical manifestations are due to stimulating antibodies directed against antigens common to the thyroid and extra-thyroid tissues (ocular and extraocular muscles, adipocytes, fibroblasts) [4].

Our patient received the diagnosis of GD in preclinical state, during health supervision and evaluation by pediatric endocrinologist for short stature. This allowed us to make early diagnosis of GD thus starting therapy as early as possible with the benefits that derive from it. Furthermore, the diagnosis of GD gave us the possibility of a personalized management of our patient with SS. In fact, one of the manifestations of the SS is the ocular involvement that also characterizes the clinical picture of the GD. Having learned that our little patient is suffering from GD, we have personalized the endocrinological and ophthalmological follow-up taking into account the possible ocular changes that the girl can develop not only because of the SS but also because of the GD.

GD diagnosis was based on detecting a suppression of serum TSH concentrations and the presence of anti-TSH receptor antibodies, according to the literature [8].

Although Graves’ disease is by far the most common cause of children’ hyperthyroidism, nevertheless other pathologies may be less frequently involved in its etiology. Hashimoto’s thyroiditis, McCune Albright syndrome, toxic thyroid adenoma, thyroid cancer, subacute thyroiditis, TSH-secreting pituitary adenomas, pituitary thyroid hormone resistance, high levels of HCG and the ingestion of exogenous thyroid hormone are other causes of thyrotoxicosis in childhood. Except for Hashimoto’s thyroiditis which can often be confused with Graves-Basedow’s disease in thyrotoxicosis phase, the other causes of children’ hyperthyroidism do not usually present differential diagnosis problems [6]. The distinguishing mark of Graves’ disease is the presence of antibodies to the TSH receptor (TSHR antibodies; TRAb) while autoimmune thyroiditis will typically have thyroid peroxidase antibodies present but not TRAb. Anti-thyroglobulin and anti-thyroperoxidase antibodies are often found to be increased (in 60–80% of cases of Graves’s disease), as a result of thyroid tissue damage [12].

Graves’s disease is predominant in girls, in children with other autoimmune diseases (linked mainly to Type 1 Diabetes, Turner’s syndrome, Down Syndrome, Di George Syndrome) and in children with a family history of autoimmune thyroid disease (AITD). Inherited forms account for 15 to 20% of cases (1st degree) [3–5, 13, 14].

A significant association between GD related hyperthyroidism and syndromic patients has already been described in literature for Turner syndrome (TS) [3] and Down syndrome (DS) [4, 5]. The prevalence of both HT and GD was reported to be significantly higher in TS girls than in the paediatric general population: respectively 10–20% vs about 1.2% for HT and 1.7–3% vs 1.07‰ for GD [15]. In DS children, HT is the most common autoimmune disease and its prevalence has been reported to be much more elevated than in age-matched patients without this condition: respectively 13–34% vs 1.3%. In DS children, also the prevalence of GD is known to be higher than in the paediatric general population: respectively 6.5‰ vs 1.07% [16].

Our patient is female with negative family history of autoimmune or thyroid disease. In addition, she has a personal negative medical history of autoimmune disease. We suspect that SS may have a role as risk factor in developing endocrine disease in pediatric age for two main factors: - the rarity of genetic condition (SS); − the rarity of endocrine disease in pediatric age (GD) in the absence of suggestive family history.

With our work we want to report - for the first time - the possibility of occurrence of Graves’ disease among the clinical manifestations of Stickler syndrome type 1.

Further diagnoses of Graves’ disease and or other immunity and endocrinological abnormalities are expected before suggesting the implementation of new clinical skills for Stickler syndrome.

In the meantime, in case of suspect, this paper may contribute to improve personalized management of this rare disease.

## Data Availability

Not applicable.

## Abbrevations

SS: Stickler syndrome
TS: Turner syndrome
DS: Down syndrome
TSH: Thyroid-stimulating hormone
MMI: Methimazole
GD: Graves’s disease
TRAb: Antibodies to the TSH receptor
AITD: Autoimmune thyroid disease
HT: Hashimoto’s thyroiditis
